# Metabolic Alterations in SARS-CoV-2 Infection and Its Implication in Kidney Dysfunction

**DOI:** 10.3389/fphys.2021.624698

**Published:** 2021-02-25

**Authors:** Magaiver Andrade Silva, Ana Ruth Paolinetti Alves da Silva, Mariana Abrantes do Amaral, Matheus Garcia Fragas, Niels Olsen Saraiva Câmara

**Affiliations:** ^1^Laboratory of Experimental and Clinical Immunology, Department of Clinical Medicine, Faculty of Medicine, Federal University of São Paulo, São Paulo, Brazil; ^2^Laboratory of Transplantation Immunobiology, Department of Immunology, Institute of Biomedical Sciences, University of São Paulo, São Paulo, Brazil

**Keywords:** metabolism, tubular epithelial cells, glycolysis, fat acid oxidation, COVID-19

## Abstract

Clinical strategies focusing on pathogen elimination are expected in an infectious-disease outbreak, such as the severe coronavirus disease 2019 (COVID-19), to avoid organ dysfunction. However, understanding the host response to viral infection is crucial to develop an effective treatment to optimize the patient’s conditions. The pathogenic viruses can promote metabolic changes during viral infection, favoring its survival, altering cell phenotype and function, and causing sustained inflammation and tissue injury. Severe acute respiratory syndrome-coronavirus 2 (SARS-CoV-2), the etiological agent of COVID-19, provokes systemic and cell metabolic changes and possibly altering lipid and glucose metabolism. Besides severe acute respiratory syndrome (SARS), SARS-CoV-2 can cause acute kidney injury, which has been associated with the severity of the disease. Although it is not clear the mechanisms whereby SARS-CoV-2 induces kidney dysfunction, it is known that the virus presents kidney tropism, namely, podocytes and proximal tubular epithelial cells. Changes in renal cell metabolism and systemic metabolic disorders are important events in kidney injury progression. Here, we explored the metabolism and its interface with SARS-CoV-2 infection and raised the perspective on metabolism disturbances as a critical event to kidney dysfunction in COVID-19.

## Introduction

The role of metabolic pathways has been little explored in the pathogenesis of several diseases. More recently, a substantial number of studies have reported that abnormal systemic or cellular metabolism is a central point in several disorders (Hotamisligil, 2006; DeBerardinis and Thompson, 2012). The metabolic functions involve different pathways, such as glycolysis, the tricarboxylic acid cycle, the pentose phosphate pathway, oxidative phosphorylation, and fatty acid oxidation, among many others, which act in an integrated manner to maintain the balance and organism homeostasis. Thereof, perturbations in these pathways are associated with the development and progression of infection and non-infection disorders (Heaton and Randall, 2010; Vastag et al., 2011; DeBerardinis and Thompson, 2012; Thai et al., 2014; Ayres, 2020).

In the last decade, an increasing number of studies aimed at investigating the crosstalk between cell metabolism and viral infection (Heaton and Randall, 2010; Vastag et al., 2011; Thai et al., 2014; Moreno-Altamirano et al., 2019; Thaker et al., 2019). These studies demonstrated that several viruses cause cell metabolic reprogramming in immune cells, including alterations on the glycolic pathway, tricarboxylic acid cycle, amino acids, and lipid synthesis (Heaton and Randall, 2010; Vastag et al., 2011; Thai et al., 2014; Moreno-Altamirano et al., 2019; Thaker et al., 2019). The fate of this metabolic perturbation is the development of viral strategies to escape from immune response and to induce severe tissue inflammation, as reported in patients infected with severe acute respiratory syndrome-coronavirus 2 (SARS-CoV-2; Huang et al., 2020; Lucas et al., 2020), the etiological agent of the coronavirus disease 2019 (COVID-19). Initial studies reported that SARS-CoV-2 causes alterations in systemic and cellular homeostasis, affecting energy metabolism (Bruzzone et al., 2020; Wu et al., 2020), and it may influence the normal function of several organs, contributing to the severity of COVID-19.

The entry of SARS-CoV-2 in host cells depends on the interaction between the Spike protein and angiotensin-converting enzyme 2 (ACE2). The Spike protein needs to be priming by specific proteases (Sungnak et al., 2020), such as TMPRSS2 (Hoffmann et al., 2020) and furin protease (Vankadari, 2020). Initially, SARS-CoV-2 infects the epithelial cells in the lungs (Zou et al., 2020). However, it can target other organs, which can considerably aggravate the clinical condition of hospitalized patients (Gupta et al., 2020), becoming COVID-19 a multiorgan disease. Kidneys are one of the main organs affected in COVID-19, resulting in elevated proteinuria, hematuria, and even acute kidney injury (AKI; Cummings et al., 2020; Hirsch et al., 2020), a severe complication in the intensive care unit associated with high mortality and morbidity (Braun et al., 2020). Studies performed in China, the United States, and the United Kingdom reported an AKI incidence of 17–43% in hospitalized patients with COVID-19, but these numbers, which are higher in patients in critical condition, range from 61 to 76% (Vijayan and Humphreys, 2020).

Histological analysis of post-mortem tissue demonstrated that viral RNA in kidneys correlates with the renal tropism of SARS-CoV-2 with early death and AKI development (Braun et al., 2020). SARS-CoV-2 preferentially infects tubular epithelial cells (Puelles et al., 2020), considered the epicenter of renal damage in the kidneys. A recent study has shown a frequency of symptoms related to kidney damage in confirmed COVID-19 patients hospitalized in Wuhan (China), where 43.9% of patients had proteinuria, and 26.7% had hematuria, increased serum creatinine levels and blood urea nitrogen, and a glomerular filtration less than 60 ml/min/1.73 m2 was observed in around ~13% of patients (Cheng et al., 2020). Other studies have also suggested that kidney function is a marker for mortality in COVID-19 patients (Brienza et al., 2020; Naicker et al., 2020; Trabulus et al., 2020).

Currently, the exact mechanisms involved in renal damage during COVID-19 have not been clear, and probably are multifactorial. Changes in systemic and cell metabolism in COVID-19 may exert an essential contribution to kidney dysfunction. In this review, we first explore the interface of metabolism and SARS-CoV-2 infection (especially at the cellular level), then raising a perspective that systemic and cellular metabolism disorders should be considered an important mechanism of renal dysfunction in COVID-19.

## Emerging Perspectives of Metabolism in COVID-19 Pathogenesis

Previous reports about virus infections demonstrated the importance of metabolism on the disease outcome. In 2003, the most critical cases of SARS happened in patients with metabolic disorders (Booth et al., 2003; Ayres, 2020), which demonstrate the importance to understand how metabolic changes could affect the course of infectious diseases as a warning of what to expect on future viral infections. Obesity, type 2 diabetes (T2D), and hypertension are related to the worst prognosis of COVID-19 (Guan et al., 2020).

It is well established that one of the critical phases of COVID-19 is the cytokine storm generated by the host response due to infection, causing an extreme inflammation process (Tay et al., 2020). Patients with previous state of chronic inflammation, as observed in most metabolic disorders (Luft et al., 2013), have more chances of presenting the cytokine storm, causing a physiological unbalance and increased health aggravation. In obesity cases, the poor condition could be due to the difficulty of ventilation associated with diaphragm excursion hampered (Burns et al., 1994); or T2D that causes decreased respiratory function, pulmonary fibrosis, and chronic obstructive pulmonary disorder (Ehrlich et al., 2010). However, the significant risk for patients with an impairment in metabolic health could be beyond respiration problems. It can also be associated with the modification of metabolism in different organs. This section described the emerging studies focusing on SARS-CoV-2 infection and its interface with energy metabolism.

### Lipid Metabolism and COVID-19

Recent studies have demonstrated the multifaceted roles of lipids in viral infection, involving lipid signaling, synthesis, and host cell metabolism modulation to subvert the protective immune response (Heaton and Randall, 2011; Murillo et al., 2015). Some studies have demonstrated that interruption in lipid synthesis impairs virus replication, suggesting that lipid pathways can represent a relevant target in the investigation of viral disorders (Merino-Ramos et al., 2016). Patients infected with SARS-CoV-2 presented altered levels of lipids. Diglycerides, free fatty acids, and triglycerides were identified in higher amounts in the fatality group (Wu et al., 2020). Furthermore, *ex vivo* and *in vitro* studies reported increased viral replication in cells with excessive intracellular lipid accumulation (Dias et al., 2020). These initial findings suggest that in SARS-CoV-2 infection, systemic and cell lipid metabolism disturbances can be critical event in COVID-19 progression.

In a recent study conducted by Thomas et al. (2020), the authors investigated the metabolic effects of SARS-CoV-2 infection by analyzing serum metabolites from patients with COVID-19 in comparison with COVID-19-negative controls (Thomas et al., 2020). The results demonstrated an increase of free fatty acids in circulation, especially in patients with high inflammatory cytokine levels (Thomas et al., 2020). Accordingly, another finding revealed alterations in a diversity of metabolites in serum of the COVID-19 patients, highlighting the expressive reduction of malic acid and glycerol 3-phosphate in fatality, severe and mild COVID-19 groups (Wu et al., 2020). Both metabolites, malic acid and glycerol 3-phosphate, are involved in energy metabolism, the first enters in tricarboxylic acid cycle in mitochondria, and the latter is a chemical intermediate in the glycolysis pathway, evidencing the alteration in metabolites that participate in human energy metabolism (Wu et al., 2020). Similar altered lipid profile was observed in SARS-CoV infection, even 12 years after recovery from the disease, patients infected with SARS-CoV revealed dysregulated levels of free fatty acids in the serum (Wu et al., 2017).

Also, SARS-CoV-2-infected human bronchial epithelial cells presented 59–65% of the differentially expressed genes related to metabolism, including 8–18% of the genes associated with lipid metabolic pathways (Ehrlich et al., 2020). However, cellular and molecular mechanisms that orchestrate lipid metabolism during SARS-CoV-2 infection are poorly described so far. Recently, it was observed the lipid bodies formation in monocytes from infected patients and *in vitro* assay of SARS-CoV-2 infection (Dias et al., 2020). The lipid bodies have been described as a source of inflammatory mediators and contribute to pathogen escape from immune system elimination (D’Avila et al., 2006, 2008; Mattos et al., 2011; Almeida et al., 2014). Dias et al. (2020) observed the colocalization of lipid bodies and SARS-CoV-2, suggesting them as a fuel for viral replication. The inhibition of lipid bodies formation reduced the viral load, cell death, and levels of inflammatory mediators. Mechanistically, the authors reported an increase in expression of transcription factor sterol regulatory element-binding protein 1 (SREBP-1) and the nuclear receptor peroxisome proliferator-activated receptor (PPARγ) after SARS-CoV-2 infection, which could be an indicative of cell reprogramming toward a lipogenic phenotype. The inhibition of the SREBP in isolated lung epithelial cells and mice infected with the Mers-CoV virus suppresses viral replication (Yuan et al., 2019), since SREBP is considered a master regulator of lipogenesis (Eberlé et al., 2004).

SARS-CoV-2 changes lipid profile in the lung epithelial cells by interfering in PPARα and PPARγ expression or activity (Ehrlich et al., 2020), culminating in lipotoxicity, which became these molecules an attractive potential therapeutic target in COVID-19 patients (Heffernan et al., 2020). PPARγ acts as a transcription factor important to CD36 expression, involved in lipid uptake (Lim et al., 2006). While PPARα is associated with control of nuclear genes encoding fatty acid oxidation enzymes (Song et al., 2010). Clinical trials using fenofibrate, a PPARα agonist, are in course in the United States as a metabolic intervention in COVID-19,^1^ which evidence the importance of lipid metabolism dysfunction in COVID-19 pathogenesis and progression.

### Glucose Metabolism and COVID-19

Besides lipid homeostasis disruption, several studies observed an increase of glycolysis activity in immune and epithelial cells from patients with COVID-19 (Codo et al., 2020; Moolamalla et al., 2020). An unmanageable blood glucose level is associated with poor diagnoses and risk of mortality, according to a study with 7,000 patients infected with coronavirus (Zhu et al., 2020).

Codo et al. (2020) demonstrated that monocytes infected with SARS-CoV-2 presented increase of ACE2 expression and viral load depending on glucose concentration. SARS-CoV-2-infected human monocytes presented a greater glycolytic capacity and reserve. The same was not observed in human monocytes infected with influenza A virus and respiratory syncytial virus (RSV). Besides, the expression of inflammatory genes (such as TNF-α, IL-6, IL-1β, INF-α, and INF-β) was glucose dose-dependent and viral replication and enhanced ACE2 expression and cytokines are decreased once the flux of glucose was blocked by 2-deoxy-D-glucose (2-DG). However, when the ATP synthase was blocked (by oligomycin), the viral load was even higher (Codo et al., 2020). Therefore, glycolysis was essential for viral replication in monocytes, being a good source of carbon, similarly observed in epithelial cells (Caco-2 cells) infected with SARS-CoV-2 (Bojkova et al., 2020; Codo et al., 2020). One factor that may explain glycolysis metabolic changes in the infected cells is the increased expression of HIF-1α, which has been implicated in the increase of glycolytic genes expression and IL-1β release (Tannahill et al., 2013). HIF-1α can regulate the activity of genes related to glucose transport and processing (LDH-A, PFKFB3, GLUT-1, PKM2), which seems to be overexpressed in monocytes from COVID-19 patients, but not at the same intensity as influenza virus and RSV-infected monocytes (Codo et al., 2020).

Monocytes and macrophages are the most common immune cell types found in the lungs of patients infected with COVID-19 recruited in response to infection and injured lung cells (Bost et al., 2020). These cells respond to infection with the exacerbated release of several inflammatory cytokines and subsidy COVID-19 outcome (Blanco-Melo et al., 2020; Tay et al., 2020). The previous study demonstrated that during SARS-CoV infection, a delay in type I interferon (IFN) expression (which is involved in the antiviral response) was associated with an inappropriate inflammatory response and lung pathology (Channappanavar et al., 2016), providing a favorable environment for viral replication and tissue injury. Similarly, in SARS-CoV-2 infection, Blanco-Melo et al. (2020) revealed that occurs a reduction of antiviral response, concomitantly with an exacerbated inflammatory response evidenced by chemokines and IL-6 production (Blanco-Melo et al., 2020). However, it was not clear whether type I IFN response was delayed, which could drive COVID-19 progression. Based on previous studies, it is plausible to suggest that modulation of glycolysis in early type I IFN response could be a strategy to increase the host defense against the virus at the beginning of infection (Zhang et al., 2019; Ayres, 2020).

Recently, it was described how glycolysis can interfere with antiviral signaling. The hexokinase-2 is the initial enzyme of glycolysis, and its activity is suggested to be dependent on physical interaction with mitochondrial antiviral-signaling protein (MAVS) and dampened IFN-I production (Zhang et al., 2019). The IFN-I production in viral infection is dependent on the virus RNA recognition in the cytosol by retinoic-acid-inducible gene I (RIG-I)-like receptor (RLR), which leads to the formation of the RIG-I-MAVS-type I IFN axis (Zhang et al., 2019). The disruption of the MAVS interaction with hexokinase-2 increases type I IFN production. Corroborating this, cells incubated with a hexokinase inhibitor increased type I IFN production, supporting the idea that the glycolysis activity interferes with the protective response in viral infection. The excessive glycolysis affects interferon production due to lactate production (one of the metabolites produced in glycolysis), which is internalized and binds to MAVS, impairing its interaction with RIG-I (Zhang et al., 2019). In SARS-CoV-2, the role of the nucleic acid sensor in the inflammation and metabolism of the different organs target by SARS-CoV-2, such as kidneys, still needs to be investigated to better understand the mechanisms in COVID-19 progression.

The kidneys are one of main organs in the regulation of systemic glucose metabolism. Because renal cells express ACE2, the kidneys become one of the main targets for SARS-CoV-2, and changes in renal metabolism may underlie the mechanisms by which SARS-CoV-2 induces AKI and aggravates clinical conditions of COVID-19 patients. Changes in systemic metabolism (as occurs in metabolic diseases) and in renal cell metabolism are reported as crucial events on decline of renal function.

## Kidney Dysfunction and Metabolism

Kidney dysfunction has long been known as an important consequence of metabolic disorders (Cohen, 1962; Joven et al., 1993). In metabolic syndrome, a clinical condition characterized by cardiovascular problems, disturbances in the metabolism of lipid and glucose have high impact on renal function (Locatelli et al., 2006; Ikee et al., 2008). Conversely, the progressive decline of the kidney function, dependent or independent of metabolic etiology, causes changes in the systemic metabolism (de Boer and Utzschneider, 2017). In physiological conditions, kidneys are responsible for up to 40% of the glucose production by gluconeogenesis, and perturbation in the metabolism of the renal cells, such as proximal tubular epithelial cells (PTECS), profoundly impacts on glucose metabolism, affecting glycolytic and gluconeogenic pathways (Legouis et al., 2020). Besides, other metabolic routes can be affected in renal injury, such as lipid and mitochondrial metabolism, starting in renal cortex, followed by medulla and plasma (Wei et al., 2014), demonstrating that altered renal energy metabolism, specifically in renal cell, is correlated with kidney injury development and it can affect systemic metabolism.

The metabolic changes occur at the cellular level and perturbations in cell energy hemostasis can lead to acute and chronic disorders. The source of energy for each renal cell type is specific, for instance, glucose is the primary energy source of podocytes, mesangial, and endothelial cells (Forbes, 2016). While PTECS supply their energy demand from fatty acid oxidation (Kang et al., 2015; Han et al., 2017). PTECS are the ones that need the most significant production of ATP because of the intense transport and reabsorption of solutes in the kidney (Bhargava and Schnellmann, 2017), and are among the renal cell types the most sensitive to renal damage.

Fatty acids act as mitochondrial substrates for oxidative metabolism in proximal tubules, and transportation of fatty acids into mitochondria is controlled by carnitine palmitoyltransferase (CPT) 1 and 2. To produce ATP from β-oxidation, fatty acids receive a coenzyme A (CoA) group through enzyme fatty acyl action synthase, resulting in a fatty acyl CoA. The fatty acyl CoA is converted to acylcarnitine by the action of CPT1 and transported to the mitochondrial inner space. In the mitochondria, acylcarnitine returns to a fatty acid acyl CoA form by the CPT2, located in mitochondrial inner membrane (O’Neill et al., 2016). In a recent study, CPT1a overexpression in renal tubule decreases renal injury by restoring mitochondrial homeostasis (Miguel et al., 2020), evidencing that mitochondria dysfunction is crucial in kidney disease development, and enzymes involved in fatty acids oxidation have a fundamental role in maintaining the mitochondria homeostasis. Kang et al. (2015) demonstrated that CPT1 inhibition reduced ATP production, causing cell death, dedifferentiation, and intracellular lipid accumulation in PTECS, which are common renal injury features. These enzymes expression is regulated by transcription factors named PPAR-α. Reduction in PPARα leads to a decreased expression of CPT1 and the peroxisomal acyl-coenzyme A oxidase 1, reflecting in the fatty acid oxidation (Kang et al., 2015). PTECS are susceptible to lipid accumulation, and a large number of studies demonstrated that excess of renal lipids causes tissue damage (Bobulescu et al., 2008; Bobulescu, 2010; Falkevall et al., 2017; Yan et al., 2018).

Mitochondrial damage and inflammatory response are classical events in AKI. The increase of mitochondria number is a protective event during experimental AKI (Tran et al., 2016). In another context, *in vitro* experiments using human PTECS stimulated with cisplatin (anticancer drug that causes nephrotoxicity and AKI development), it was observed a reduction in the mitochondrial fatty acid oxidation (Maekawa et al., 2019), leading to lipid accumulation. The lipid excess induces reactive oxygen species production, apoptosis, inflammation, profibrotic factors release, and organelle damage (Weinberg, 2006; Bobulescu, 2010). In addition, lipotoxicity can occur due to the impact of hypoxia on them (Ruidera et al., 1988; Bobulescu et al., 2008), which is one of the mechanisms that potentially causes tubular damage. Based on these findings, renal lipotoxicity may be contribute to kidney damage in COVID-19 patients, since individuals with COVID-19 present respiratory insufficiency that leads them to hypoxemia, worsening peripheral tissue ischemia (Del Vecchio and Locatelli, 2020).

Besides the alterations in lipid metabolism observed in renal dysfunction, the metabolism of glucose can also be altered leading to deleterious events. During AKI, the PTECs present an increased glycolytic profile, and this change is exceptionally critical in their physiology during recovery after AKI. In ischemia-reperfusion injury, the metabolic switch occurs early during regeneration after insult and tubules become atrophic. However, even regenerating tubules present increased glycolytic enzyme expression, and this irreversibility of metabolic profile led the cell to hypoxia and induced the profibrotic signaling (Lan et al., 2016), which can contribute to the progression from AKI to chronic pathology. In line with this, it was observed an increase in glycolytic profile in experimental and clinical AKI, in contrast with the reduction of gluconeogenesis (Legouis et al., 2020). It was observed that rate-limiting gluconeogenesis enzymes were decreased during the early phase following ischemia-reperfusion injury, but the expression of glycolytic enzymes was increased. The reduction of renal gluconeogenesis can contribute to hypoglycemia in stress conditions, compromising the systemic metabolism and contributing to worsening patient condition. Metabolic reprogramming of glucose metabolism during AKI was associated with mortality, as reported by Legouis et al. (2020). COVID-19 patients with metabolic disorders have a worsening of the clinical condition associated with acute kidney disease, which suggests that dysfunction in systemic metabolism may contribute to renal injury in COVID-19.

## Metabolism and SARS-CoV-2: Possible Implications on Renal Injury Development

Currently, it is already known that SARS-CoV-2 can change host metabolism. The consequences of the metabolic alteration in COVID-19 for organ functions, especially the kidneys, are poorly described. An investigation with 33 diagnosed patients with COVID-19 in comparison with COVID-19-negative individuals demonstrated that altered metabolite levels of the fatty acid and tryptophan metabolism in infected patients were correlated with clinical markers of inflammation (IL-6 and C-reactive protein) and renal function (BUN and creatinine; Thomas et al., 2020). Besides, *in vitro* studies demonstrated the SARS-CoV-2 potential of modulating the lipid metabolism in monocytes and lung epithelial cells (Dias et al., 2020). The abnormal metabolism functioning is critical for renal injury development, which makes systemic and cellular metabolism in COVID-19 an exciting issue of investigation for further studies in the context of renal injury.

A retrospective analysis found that patients with COVID-19 presented altered blood glucose levels (hypoglycemia and hyperglycemia) in the course of disease accompanied by poor outcomes, including AKI. In the patient’s group that achieves a mean glycemia of 140 mg/ml, 24% of them experienced at least one episode of hypoglycemia (blood glucose levels below 70 mg/dl) and presented an increased risk of AKI and mortality. However, the exact cause of hypoglycemia in these patients is unknown (Klonoff et al., 2020). Legouis et al. (2020) observed that gluconeogenesis is impaired in renal PTECs in clinical and experimental AKI. In this study, the author verified the increase of glycolytic enzymes and reduction of gluconeogenesis, demonstrating that the glucose metabolism reprogramming in renal PTECs had an effect on systemic levels of glucose and was correlated with patient mortality. The high death rates in COVID-19 associated to AKI may be due to alterations in the metabolism of PTECs caused by systemic or direct infection of renal cells by SARS-CoV-2.

The molecular mechanisms involved in metabolic dysfunction in COVID-19 are still sparsely described. An *in silico* study demonstrated the interaction of the spike protein (S protein) from SARS-CoV-2 with human innate immune receptor, named Toll-like receptors (TLRs), which are a type of pattern recognition receptors. Molecular docking revealed the potential binding of the S protein of SARS-CoV-2 to TLR-1, -4, and -6, presenting binding energy value of −57.3, −120.2, and −68.4, indicating that TLR4 has a high affinity to S protein following TLR6 and TLR1 (Choudhury and Mukherjee, 2020). TLR4 has been associated with inflammatory conditions and its activation induces metabolic changes in macrophages and dendritic cells, altering mitochondrial, lipid, and glycolytic homeostasis (Everts et al., 2014; Perrin-Cocon et al., 2018; Lauterbach et al., 2019). In renal context, TLR4 activation induces severe inflammation and AKI (Cenedeze et al., 2007; Andrade-Silva et al., 2018). However, whether the metabolic dysfunction of SARS-CoV-2-infected patients can be associated with TLRs signaling in the kidneys remains unclear.

Therefore, further studies aiming at cellular and molecular mechanisms in SARS-CoV-2 infection and kidney pathology are urgent topics of investigation.

**Figure 1:**
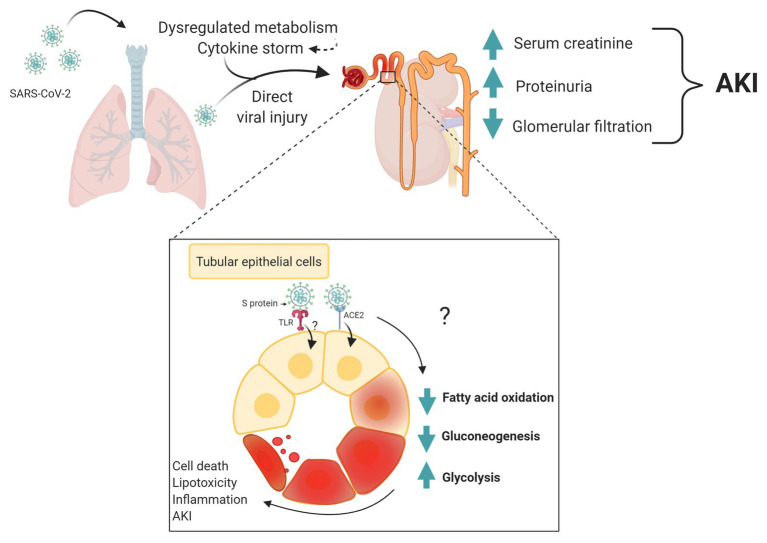
Metabolic changes as a potential mechanism of acute kidney dysfunction in coronavirus disease 2019 (COVID-19). Severe acute respiratory syndrome-coronavirus 2 (SARS-CoV-2) infects lung epithelial and lead to extrapulmonary systemic hyperinflammation. Products of inflammation and metabolites may cause renal metabolic dysregulation. This affects could be triggered by direct infection of kidney cells by SARS-CoV-2. Patter recognition receptor, such Toll-like receptor (TLR) may be important molecular mechanism activate by virus proteins and triggering changes in tubular epithelial cell metabolism and inflammation. Both scenarios, systemic and/or direct changes in metabolism may be associated to acute kidney injury in COVID-19. Created with www.BioRender.com.

A proposal mechanism for the acute renal dysfunction development in COVID-19 and its interface with metabolism is shown in Figure 1.

## Final Remarks and Perspectives

The interface between cell metabolism and inflammation is an emerging topic in immune and non-immune disorders. Disturbances in metabolism are associated with inflammation and targeting host cellular metabolism in severe disease is undoubted point to be considered in clinical management of the affected patients. Urgently, the world hopes for solutions for COVID-19 complications. Undoubtedly, the kidney represents a critical organ that, when affected, can be determinative in morbidity and mortality of COVID-19 patients. The focus on COVID-19 should be directed not only on pathogen elimination but also on the physiological alterations during infectious processes, such as systemic and cellular metabolism changes and more studies to clear how metabolism can be determinative in tissue injury progression. Currently, little is known about the long-term effects of SARS-CoV-2, but another species of coronavirus already demonstrated the potential to cause metabolic disorders even many years after the patient recovery of infection. Understanding the systemic and intracellular metabolic alterations and its consequences in COVID-19 will help to design better pharmacological therapy, repurposing drugs used in metabolic disorders aiming at improvement of hospitalized patient clinical conditions, and reduction of death rates or sequelae.

## Author Contributions

MA-S and NC conceived the concept of the manuscript. All authors contributed to the literature review and writing of the manuscript and approved for its publication.

### Conflict of Interest

The authors declare that the research was conducted in the absence of any commercial or financial relationships that could be construed as a potential conflict of interest.

## Footnotes

^1^clinicaltrials.gov/ct2/show/NCT04517396
